# Vienna Letter

**Published:** 1882-06

**Authors:** W. T. Belfield

**Affiliations:** Vienna


### ARTICLE V.

VIENNA, MAY 2, 1882.

EDITORS MEDICAL JOURNAL AND EXAMINER:—The topic of the month has been the demonstration by Dr. Robert Koch, before the Physiological Society in Berlin, of bacilli, which he asserts, after a series of observations and experiments extending over some two years, to be the direct exciting cause of phthisis and tuberculosis. His statements may be summarized as follows:

Tuberculosis, in the most comprehensive sense of the term, including miliary tuberculosis, cheesy pneumonia, etc., is a parasitic disease, induced by a bacillus similar to that of lepra. By means of special staining methods (first with methylviolet and then vesuvin) Koch always detected the characteristic motionless rods, especially in places where the process was most recent; the rods were less numerous around the giant-cells. He succeeded, moreover, in cultivating the bacillus, quite free from all admixtures, in blood-serum and gelatine. It grows very slowly, and only under a temperature between 30 and 40 degrees Cent.

From these artificially cultivated bacilli (the cultivation extended over some six months, with constant transfer to fresh glasses) he inoculated a large number of animals—guinea-pigs, rabbits, rats, dogs—sometimes by means of simple incision in the skin; sometimes in the anterior chamber of the eye, sometimes by injection into the circulation. The result was almost invariably the production of acute tuberculosis—in many cases, further, the induction of cheesy transformations of tubercle. A number of specimens were demonstrated.

The alleged discovery of the tubercle parasite is by no means a novelty. Some time ago Aufrecht announced that he had seen them, and Klebs, who seems to have a mania for such things, was even earlier in the field; but these isolated observations, unsupported by experimental or other evidence, were classed with the numerous similar "discoveries" of pathogenic bacteria, in which recent literature abounds. Koch's researches, however, are so extensive and accurate, so thorough and convincing, and his previous investigations in the same direction have been so admirable, that his statements cannot be ignored, and we may expect that the question will now receive thorough investigation by competent hands.

It is a little amusing that Baumgarten, in Königsberg, a few days after Koch's demonstration in Berlin, published the discovery of bacteria in tuberculosis artificially produced in rabbits—a discovery made several weeks before Koch's publication. His methods were entirely different from Koch's, and a little comparative investigation showed that Baumgarten's bacteria were entirely distinct in size and shape from Koch's. Moreover, both Baumgarten and Koch refnse most decidedly to allow any relationship between their bacteria and the stuff which Klebs, Schueller and Aufrecht regarded as the parasitic agent in tuberculosis. Up to date there are, therefore, at least three entries for the tuberculosis handicap, with large odds on Koch's parasites against the field.

At the recent Congress of German physicians at Wiesbaden, Koch's address (essentially the same as in Berlin) called forth an animated discussion. Aufrecht, of Magdeburg, remarked that the question of tuberculosis as infectious disease could not be considered as definitely settled by this discovery, and that until a definite solution was reached, social and hygienic conditions must continue to play an important part in the conception as to the etiology of the disease. Seitz, of Munich, declared himself opposed to the unconditional infection theory. The assumption that tuberculosis is an infectious disease, due to the inhalation of bacteria, is by no means so good an explanation of the observed phenomena as the older view as to etiology. How, he asked, shall we explain the unquestioned heredity of the disease? and how the "phthisical habit" which so often heralds the coming consumption? There must be assumed, not simply an infection, but also such constitutional conditions as favor the development of the infecting material. Rühle, of Bonn, emphasized the fact that phthisis and tuberculosis are by no means to be regarded as identical. That phthisis induces tuberculosis is proven, but it remains to be demonstrated that tuberculosis bears a causal relation to phthisis.

Jürgensen (Tübingen) was favorably disposed toward Koch's views. His experience in a region comparatively free from tuberculosis has led him to believe that a bronchial mucous membrane, in a catarrhal condition in consequence of enfeebled heart's action (in old people generally, and in children with catarrhal pneumonia) was a by no means favorable soil for the reception of the tubercle parasite. Klebs, of Zürich, adopted Koch's views, of course; he expressed the hope that the question of heredity (whether, namely, the transmission occurs directly from mother to fœtus, or after birth, by means of the milk) will soon be thoroughly studied.

Bagnisky, of Berlin, thinks that the question raised by Seitz and Rühle was solved by observations on children; that the identity of phthisis and tuberculosis was not to be ignored; the variability in the phenomena of the two depends upon the nature of the soil infected. It is well known that little children rarely exhibit phthisis in characteristic form; they become tuberculous. The explanation is that the child's lung, thanks to the relatively greater strength of the right heart in early life, is far more succulent than in more advanced years; for in adult life, the left heart so far excels the right in power, that the lung is less succulent; hence the same infectious material can induce different pathological conditions at different periods in life, or in different individuals of the same age.

The iodoform war continues without abatement. One of the most ferocious among the numerous assailants is Kocher, of Bern, who thinks that the use of iodoform should be forbidden, in the interest of public safety, either by sanitary authorities or by the surgical congress. He says, moreover, that the subnitrate of bismuth accomplishes all the good results claimed for iodoform, and is at the same time cheaper and odorless. He promises a detailed account of his experience with this substance at some time in the near future.

König supplements his previous report with sixteen additional cases of intoxication from iodoform dressings. These tend to confirm his deductions from the previous cases. Eleven of the sixteen patients were more than thirty-five years old; seven over fifty. The five fatal cases occurred in patients aged seventy-two, fifty-six, fifty-eight, fifty-two and thirty-seven years respectively. In one he observed an exanthem closely simulating scarlet-fever rash.

One of these new cases is worthy of note. The operation was a resection of the knee. The first bandage—iodoform, quantity not stated—was allowed to remain six days; subsequently the bandages were changed every second or third day. Immediately after the operation the pulse increased in frequency from 80 to 120; during the first sixteen days it varied between 100 and 150; in the third week it was less frequent; in the fourth and fifth weeks 130 to 160, sometimes not to be counted. The temperature was constantly high—in the fourth and fifth weeks over 40° C.; then chills, hallucinations, loss of appetite, emaciation, were observed; the wound became painful. Under the conviction that pyæmia existed, the limb was amputated in the fifth week after the resection. Union occurred promptly under iodoform dressings, and the patient made a satisfactory recovery. In the amputated member there was found no pus, neither in the soft parts nor in the bones, but a *lump of iodoform between the resected bone surfaces.*

The discussion in the Berlin society as to the results of nerve-stretching for the cure of tabes dorsalis brings but little new. Several cases which Langenbeck had considered successful have been pronounced by others failures. Israel gives his own experience; he has seen marked, but only temporary improvement. One case was noteworthy—a case of advanced tabes, with general analgesia and deficient tactile sensibility over the entire body. The right sciatic nerve was stretched. Two days later the analgesia had vanished. The power of localization had returned. The patient could locate correctly needle-pricks, and could distinguish the head from the point. The girdle sensation had ceased. The gait was much improved; the man could stand with closed eyes without reeling noticeably, etc. The improvement still continues, but Israel expresses no confidence as to its permanence.

Hahn has operated in twenty-five cases, and summarizes his experience as follows: "I have achieved temporary improvement in cases of neuralgia, tonic and clonic spasms, epileptiform attacks of peripheral origin. In cases of trismus, tetanus and tabes, the operation has, in my hands, always failed."

Westphal deduces from the discussion that not a single case of tabes dorsalis has been cured by nerve-stretching; it is even doubtful if particular symptoms, especially the pains, can be bettered for any considerable time; certainly, in many cases, this symptom has not been in the least improved. He thinks that nerve-stretching can do no more than internal medication, electricity, etc.

Billroth's assistant recently reminded the medical public that the woman whose pylorus he extirpated in April, 1881, still lives, is in excellent health, and exhibits no signs of recurrence of the cancer. Billroth's own case, operated on six months ago, is in a condition equally satisfactory. A patient upon whom Czerny performed this operation some months ago, has gained forty pounds in weight since. Billroth and his school are evidently determined to prove the possibility of permanent immunity from the disease through this operation.

W. T. BELFIELD.

---

A REPLY TO DR. A. B. PALMER'S BOOK ON "HOMŒOPATHY—WHAT IS IT?"—Dr. James. H. Patton, of Richmond, Va, will commence, in the April number of the *Southern Clinic* a series of papers, which will be a review and reply to Dr. Palmer's work.

Dr. Palmer's book will be methodically taken up and answered chapter by chapter. Dr. Patton is a graduate in regular medicine, as also of the homœopathic school, a prominent physician, and a trenchant writer.

